# Immediate determination of ACPA and rheumatoid factor - a novel point of care test for detection of anti-MCV antibodies and rheumatoid factor using a lateral-flow immunoassay

**DOI:** 10.1186/ar3057

**Published:** 2010-06-22

**Authors:** Franziska Renger, Holger Bang, Eugen Feist, Gert Fredenhagen, Alexander Natusch, Marina Backhaus, Gerd-R Burmester, Karl Egerer

**Affiliations:** 1 Department of Rheumatology and Clinical Immunology, Charité - Universitätsmedizin Berlin, Charitéplatz 1, 10117 Berlin, Germany; 2 ORGENTEC Diagnostika GmbH, Carl-Zeiss-Str. 49, 55129 Mainz, Germany; 3 Department of Rheumatology, Immanuel Krankenhaus, Karower Straße 11, 13125 Berlin-Buch, Germany

## Abstract

**Introduction:**

Autoantibodies against mutated and citrullinated vimentin (MCV) represent a novel diagnostic marker for rheumatoid arthritis (RA). Recently, an increased sensitivity for anti-MCV compared to autoantibodies against cyclic citrullinated peptides (anti-CCP2) was shown in cohorts of patients with early RA and established disease.

The aim of this study was to develop and evaluate a point of care test (POCT) for detection of anti-MCV antibodies immediately at the first visit or at the bed side.

**Methods:**

A lateral-flow immunoassay was developed for simultaneous detection of anti-MCV antibodies and rheumatoid factor (RF-IgG) and evaluated in a prospective setting. Analyses were performed from whole blood samples of patients with seropositive RA (n = 108), seronegative RA as well as other rheumatic disorders (n = 122), and healthy blood donors (n = 200) and compared to detection via ELISA.

**Results:**

Using the POCT, anti-MCV antibodies were detected in 54.6% and RF-IgG in 56.5% of patients with RA. Specificity was 99.1% for anti-MCV antibodies and 91.2% for RF-IgG. Compared to ELISA's results, POCT sensitivity was 69.3% for anti-MCV and 55.6% for RF-IgG, specificity was 99.7% and 97.2%, respectively.

**Conclusions:**

This POCT for detection of anti-MCV antibodies and RF-IgG provides high specificity for the diagnosis of RA and is useful in clinical practice due to its simplicity and its reliable performance. This test can greatly improve a timely management of RA and may help in screening patients with suspected RA in non-specialized settings prompting early referrals.

## Introduction

Rheumatoid arthritis (RA) is the most common chronic autoimmune arthritis worldwide leading to disability and substantial economic costs [1,2]. For improving the overall outcome and to prevent irreversible joint damages, early diagnosis and therapy are crucial. However, the initial clinical signs of RA are often non-characteristic, rather resembling undifferentiated arthritis. Detection of autoantibodies against citrullinated protein/peptide antigens (ACPA) substantially improved our diagnostic repertoire providing moderate sensitivity and high specificity for early-RA. Recently, we identified a novel antigenic isoform of vimentin in patients with rheumatoid arthritis, which was modified by citrullination and mutation (MCV) [3]. Subsequently, several investigators in different cohorts of patients with rheumatoid arthritis reported on diagnostic performance for anti-MCV antibody testing ranging from 69 to 82% for sensitivity and reaching 81 to 98% for specificity [3-12].

To further facilitate ACPA testing, a point of care test (POCT) was developed for a rapid and combined detection of rheumatoid factor (RF) and anti-MCV-antibodies. This rapid test can be performed from one single drop of whole blood and does not require any additional equipment. To evaluate the diagnostic performance of this novel POCT for RF-IgG and anti-MCV-antibodies and compare it with established procedures, a prospective study was performed in patients with RA.

## Materials and methods

### Patients

In this study, 108 patients with (thus far) seropositive RA fulfilling the revised ACR criteria, 122 patients with seronegative RA and other rheumatic disorders, and 200 healthy blood donors were analyzed for anti-MCV and RF-IgG seropositivity using the POCT as well as commercially available ELISAs (See Table 1 for patients' characteristics).

**Table 1:** Patients' characteristics

	RA patients	Control group	Healthy blood donors
total	108	122	200

female/male (n)	87/21	84/38	139/61

age mean (SD)	57.9 (13.8)	57.1 (14)	47.4 (19.1)

SD, standard deviation

Main diagnoses in the control group were ankylosing spondylitis (n = 21), psoriatic arthritis (n = 21), seronegative course of rheumatoid arthritis (n = 20) and Sjögrens' syndrome (n = 9), polymyalgia rheumatica (n = 8), systemic vasculitis (n = 7), systemic lupus erythematosus (n = 7), Lyme borreliosis (n = 6) and osteoarthritis (n = 6) (all patient diagnoses are listed in Additional file 1).

All patients were recruited from the in- and outpatient clinics of the Department of Rheumatology at the Charité-Universitätsmedizin Berlin and at the Rheumaklinik Berlin-Buch. The study was approved by the local Ethics Committee, and blood samples were obtained after written informed consent.

### Lateral-flow immunochromatographic device

Lateral-flow immunochromatographic assay (LFIA) was manufactured as double antigen direct sandwich assay. Devices (DCN, Carlsbad, CA, USA) for testing of up to 10 μl of biological samples were produced by mounting a nitrocellulose membrane (Thickness, 205 ± 1 μm) (Millipore, Billerica, MA, USA) to a plastic support. Purified recombinant MCV and purified Fc-part of human immunoglobulin (approximately 1 mg/ml each) were striped in two test line (MCV and RF) positions, while protein L (0.5 mg/ml) (Sigma, St. Louis, MO, USA) was striped in the control line position *C*. Gold particles (40 nm, British BioCell International), were individually conjugated to goat anti-human IgG and IgM (Dianova, Hamburg, Germany) and mixed. Anti-human immunoglobulin colloidal gold conjugate was dispensed onto a conjugate pad (Arista Biologicals, Allentown, PA, USA). The conjugate pad was then affixed to the test strip by overlapping the nitrocellulose membrane at its proximal end. The assembly was completed by addition of a sample pad onto the conjugate pad. Assay buffer consists of 20 mM Tris, 0.01% sodium azide, 250 mM NaCl, 0.05% Tween 20. Test performance was stable for at least 24 months after manufacture by storage at room temperature.

### Direct antibody sandwich format

A blood drop (approximately 20 μl) was placed in the *sample port *at *A *on the device. After adding six drops of assay buffer into the buffer port *B*, patients' antibodies migrated down to the nitrocellulose membrane by capillary action. At the test line *T *anti-MCV or RF bound to their respective immobilized antigens. By adding an assay buffer, the anti-human IgG gold conjugate was resuspended, and after migration on the nitrocellulose membrane indicated the autoantibody-antigen complexes formed as a red line. Non-MCV and RF specific antibodies migrated to the control line *C *and were visualized by gold-conjugated anti-human IgG.

During development of the assay, the amount of gold-conjugated anti-human IgG, the number of conjugated colloidal gold particles, and the amount of anti-human IgG were empirically titrated to yield a distinct line at the test positions using a serum sample with a reactivity of approximately 100 U/ml in both standardized anti-MCV and RF-ELISA (Orgentec, Mainz, Germany).

**Figure 1:**
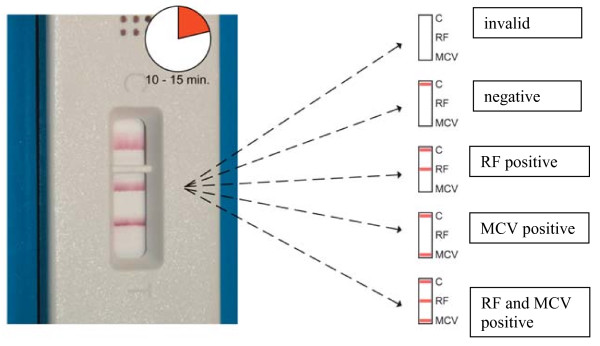
**Results**.

The ratio of applied antigens and serum anti-MCV antibodies and/or RF was such that monodentate binding of autoantibodies to the epitopes was favoured on the basis of steric and other conditions. Subsequently, bidentate antibody binding was favoured at the two *test lines*, an anti-MCV and a RF-IgG binding sites, due to the extremely high concentration of antigens (approximately 2 mg/ml). Once optimized, this process became independent of the concentration of serum anti-MCV and RF. The colour formation for all reactions was completed after 10 to 15 minutes. The device provides an integrated control system indicating correct test performance or invalid test results. See Figure 1 for possible result constellation.

Serum samples and whole blood samples were run in the LFIA device, and the values were compared to ELISA-derived anti-MCV and RF concentrations, respectively. Results were dichotomized on the basis of being above or below the limit of quantification of the ELISA (cut-off anti-MCV 40 U/ml, cut-off RF-IgG 30 U/ml).

### ELISA

Anti-MCV antibodies (cut-off 20 U/ml) and RF-IgG (cut-off 20 U/ml) were determined by an ELISA (Orgentec).

### Statistics

Sensitivity, specificity and predictive values were calculated according to the appropriate formula. Sensitivity was exclusively calculated within the RA group. Specificity was calculated against rheumatic diseases and a healthy control group. In this study, prevalence was considered as the ratio of seropositive RA patients against all patients with rheumatic diseases (n = 230).

## Results

Whole blood samples of 108 patients with seropositive RA underwent LFIA testing and showed 59 positive anti-MCV results and 61 positive RF-IgG results, reflecting a diagnostic sensitivity of 54.6% for anti-MCV and 56.5% for RF-IgG (Table 2). The positive POCT results were confirmed by ELISA in 98.3% and 95.1% of the cases, respectively. Testing 122 patients with other rheumatic disorders led to no positive anti-MCV results in POCT and 10 positive RF-IgG samples (Table 3). In contrast, ELISA for anti-MCV antibodies was positive with 4 and for RF with 35 patients. Therefore, specificity for MCV was 99.1% and for RF 91.2% using the POCT. Analysis of 200 healthy blood donors revealed 3 anti-MCV positive and 16 RF-IgG positive results, which were confirmed by ELISA in 100% and 93.7% of the cases, respectively (Tables 2 and 3).

**Table 2:** Results of Anti-MCV and RF IgG testing using POCT in comparison to ELISA

POCT	Anti-MCV	RF IgG
positive (n)	62	87

negative (n)	368	343

sensitivity relating to diagnosis (%)	54.6	56.5

specifity relating to diagnosis (%)	99.1	91.2

PPV relating to diagnosis (%)	100	

NPV relating to diagnosis (%)	71.3	

sensitivity relating to ELISA (%)	69.3	55.6

specifity relating to ELISA (%)	99.7	97.2

PPV, positive predictive value; NPV, negative predictive value

**Table 3:** Results of POCT for different patients' groups and controls

Results	RA (n)	Control group (n)	Blood donors (n)	Total (n)
**MCV+ RF IgG+**	46	0	3	49

**MCV+ RF IgG-**	13	0	0	13

**MCV- RF IgG+**	15	10	13	38

**MCV- RF IgG-**	34	112	184	330

**total**	108	122	200	430

The positive predictive value of anti-MCV positivity regarding the diagnosis RA was 100% and the negative predictive value was 71.3% using the POCT. Having defined the ELISA results as the gold standard these results led to an overall sensitivity of 69.3% for detection of anti MCV antibodies and of 55.6% for RF-IgG as well as to a specificity of 99.7% for MCV and of 97.2% for RF-IgG. Overall, correlation between both methods was 93% regarding detection of anti-MCV antibodies and 83% regarding detection of RF-IgG in all 430 samples. There was no invalid test result among all 430 samples.

## Discussion

At an early undifferentiated stage of disease diagnosing RA can be difficult and challenging as clinical manifestation may appear oligosymptomatic, intermittent, or asymmetric, not yet fulfilling the current classification criteria of the disease. However, major joint damage and loss of function occur during the first months and years of the disease. Thus, early diagnosis and consecutive treatment with disease modifying anti-rheumatic drugs (DMARDs) are essential in order to manage rheumatoid arthritis successfully. Former studies investigating lag time from symptom onset to administration of antirheumatic drugs showed that the greatest time loss occurs either during the diagnosis of RA or the time until referral to a rheumatologist. Once diagnosis was made or patients were seen by a rheumatologist, antirheumatic therapy was administered within a few weeks only [13,14]. Therefore, a POCT providing immediate results for a highly specific marker for RA such as anti-MCV antibodies applied at the primary care doctor's practice with a patient with unclear joint symptoms and suspected RA can accelerate referral to the specialist leading to more detailed laboratory tests, earlier diagnosis and therapy. Moreover, in rural settings or in developing countries more elaborate test systems such as ELISA may not be available or their results may take too long to be taken into account.

In this study, a mid-range sensitivity and excellent diagnostic specificity were documented for the simultaneous detection of anti-MCV antibodies and RF-IgG using POCT. As a major technical difference to established immunoassays, this POCT is based on the ability to detect anti-MCV antibodies and RF from one single drop of whole blood. It does not require washing steps or special equipment. Results come within 15 minutes. The test result can be evaluated visually, typically by recognition of up to two test lines (RF and MCV) and a control line for correct test performance. Therefore, this test might be extremely useful in clinical practice due to simple and reliable performance. Moreover, by providing a high specificity for RA, this test allows an excellent point of care testing. Although false positive results are rare, positive reactivity in POCT should be confirmed by using a standard immunoassay such as an ELISA.

The POCT we investigated in this study is the first POCT testing for anti-MCV antibodies and RF-IgG. RF-IgG, although of limited clinical value, was chosen for reasons of technical feasibility of the device. A second generation POCT will test anti-MCV and RF-IgM, the only laboratory classification criterion for RA to date. A clinical sensitivity of 54.6% for the detection of anti-MCV may be due to the fact that almost all patients have been under treatment which might act on the detectability of the antibodies. ELISA's analyses showed 75% sensitivity; this gap is most likely explained by reason of slightly different epitopes. In 2008, Snijders *et al*. reported on a POCT testing for anti-CCP2 antibodies from capillary blood of 109 RA patients showing 95% sensitivity and 95% specificity regarding ELISA's results [15].

## Conclusions

Recent developments reflect the need for simple and quick tools in helping to spot patients at high risk for an aggressive course of disease in order to optimize management of RA.

In summary, this novel rapid test system for the detection of disease specific autoantibodies can significantly improve a timely management of RA and may help in screening patients with suspected RA, prompting early referrals even in non-specialized settings.

## Abbreviations

ACPA: autoantibodies against citrullinated protein/peptide antigens; anti-MCV: autoantibodies against mutated and citrullinated vimentin; anti-CCP: autoantibodies against cyclic citrullinated peptides; DMARDs: disease modifying anti-rheumatic drugs; ELISA: enzyme-linked immunosorbent assay; LFIA: lateral-flow Immunoassay; POCT: point of care test; RA: rheumatoid arthritis; RF: rheumatoid factor

## Competing interests

G Fredenhagen and H Bang are employees of Orgentec Diagnostica, a company which sells autoimmune test systems. E Feist received honoraria from Orgentec. K Egerer received grants (AiF cooperation research project sponsored by BMWi) from Orgentec. H Bang is the inventor and patent holder of MCV, one of the antigens used for the POCT.

Orgentec gave financial support but had no influence on the planning of the study, analysis of data or manuscript preparation.

## Authors' contributions

FR performed blood collection, data acquisition, statistics, and created graphics and partially wrote the manuscript. HB and GF developed the method and provided technical details. EF contributed to data acquisition and partially wrote the manuscript. AN and MB contributed to data acquisition. GRB was involved in designing the study, drafting the manuscript and critically revising it. KE, as the last and responsible author, initiated this study and controlled the work. KE reviewed the manuscript. All authors approved the final manuscript.

## Supplementary Material

Additional file 1 **Supplementary table**. Diagnoses of the control groups. Click here for file
