# Supplementary material for: Immediate determination of ACPA and rheumatoid factor - a novel point of care test for detection of anti-MCV antibodies and rheumatoid factor using a lateral-flow immunoassay
Source: Arthritis Res Ther. 2010 Jun 22;12(3):R120. doi: 10.1186/ar3057 (PMC2911914; doi:10.1186/ar3057)
Supplement: Additional file 1 — Supplementary table. Diagnoses of the control groups. [file ar3057-S1.RTF]

Supplementary Material

Table 4: Diagnosis of the control groups

Diagnosis	Number of Patients	
Psoriasis  - Arthritis	21	
Spondylitis ankylosans (M. Bechterew)	21	
Seronegative Rheumatoid Arthritis	20	
Sjögren's – Syndrom	9	
Polymyalgia rheumatica	8	
Systemic Lupus erythematosus	7	
Systemic Vasculitis	7	
Lyme borreliosis	6	
Osteoarthritis	6	
Gout	2	
Scleroderma	3	
Oligoarthritis	2	
Mixed connective tissue disease	2	
Undifferentiated connective tissue disease	1	
Uveitis and Arthritis	1	
Juvenile ideopathic Arthritis	1	
Zöliacia and Arthritis	1	
Colitis ulcerosa and Arthritis	1	
Reaktive Arthritis	1	
SAPHO – Syndrome	1	
Undifferentiated Sacroiliitis	1	
